# Spina Bifida Occulta

**Published:** 1893-02

**Authors:** A. Bowen

**Affiliations:** Nebraska City, Neb.


### SPINA BIFIDA OCCULTA.

BY A. BOWEN, M. D. NEBRASKA CITY, NEB.

On the 25th of January, 1892, was called to J. S., a young man whom, though wasted by a month's severe sickness, gave promise by his muscular endowment of the eulogium I had heard a farmer of my acquaintance bestow upon him, that he was the most profitable hand he had ever hired.

I found him with an irritable pulse of 98, face bedewed with perspiration and many of the signs of hectic fever with great prostration and all the symptoms justifying the prognosis of the medical man who had preceded me and who said to his mother that he would not live ten days. He was 24 years of age, and wasted as he was, it was a laborious task for his friends to support him 20 inches above the bed, while I examined his back where all his complaint centered. I found it one vast burrowing abscess from his shoulder blades to his hips. It had been lanced at two points not far from the spine, without any attempt at a valvular opening and without any regard to antisepsis. It needed but a very little encouragement from a probe to evacuate from 15 to 20 ounces of fluid, part of which was unmistakably pus, and part seemed something else and much thinner. I found a yielding elastic protuberance at the foramen magnum, about the size and shape of half an egg, which his mother insisted never showed itself until he was after ten years of age.

Convinced from the first that we must have more available means of suspending him above the bed for the purpose of dressing and examining his back, I bethought myself of a block and tackle, and it answered an excellent purpose for 38 days. Feeling assured that the wonderfully profuse secretion from his back jeopardized his life, I sent into every part of the abscess by means of a small glass syringe a 4 per cent. solution of carbolic acid, and as I found it immediately effective, I reduced the strength from day to day till I was soon using only a 2 per cent. solution, and the secretion of pus from all the apertures did not amount to one-fourth of a tea-spoonful per day, and the thinner fluid which exuded from two of what were now ulcers, at the points which had been lanced, did not amount to more than one-fourth of a teaspoonful. I was satisfied that the thinner fluid had been intended by nature for lubricating the cavity of the spinal cord.

I once had born into my hands an ordinary case of spina bifida, in which, before I left the house, I had to protect the exceedingly thin membrane with a piece of oiled silk, and as long as the child lived there was an exudation of similar fluid.

I felt under many obligations to the ladies in his neighborhood for supplying him with delicacies from their own tables, till a natural, healthy appetite rendered it unnecessary. For two months now the patient has sat upon a chair for me to dress his back, and though I talk encouragingly to him, it seems almost impossible to cover the ulcer with cuticle from which exudes the thin fluid. He is industrious and saws all the wood for family use. The bed sores (at one time very threatening) have all healed, and he seems in a fair way for perfect recovery, but I shall always give a guarded prognosis of his future. I cannot learn of any blow or strain his back has received at any time, but I learn from measurement that his left arm is a full inch smaller than his right arm, and by palpation I learn that from about the 5th cervical vertebra clear down through the dorsal and into the lumbar, I cannot distinguish any projections for any of the vertebra below the 5th cervical. He commenced complaining of severe pain about the foramen magnum about January 1st, and the tumor at that point increased in fulness at that time, though now apparently empty. I consider his ailment as a manifestation of la grippe. When tired, he complains of a tingling sensation in the third and fourth fingers of his left hand. I view the primary lesion of la grippe as a neurosis, followed in many instances by sequela threatening to life through pulmonary or bronchial disease.

### IMPURE BROMIDES.

Helbing's Pharmacological Record has an important statement concerning the undue proportions of potassium chlorate that are found in the bromides. An examination made by Helbing and Passmore show that it is a serious matter to buy the potash salt at the present time without having it carefully analyzed as to the percentage of chlorides it may contain. The importance of purity in a drug of this nature is very great and will receive the earnest heed of neurologists everywhere. —*Journal American Medical Association.*

[PEACOCK'S BROMIDES are of known purity, and should be used when bromides are indicated, as they are the *only* preparation of *Chemically Pure* Bromides on the market.]

